# Cerebrovascular Reactivity to Acetazolamide in Stable COPD Patients

**DOI:** 10.3390/jcm14238535

**Published:** 2025-12-01

**Authors:** Péter Siró, Regina Szabó-Szűcs, Viktória Dudás, Ildikó Horváth, Béla Fülesdi, Attila Vaskó

**Affiliations:** 1 Department of Anesthesiology and Intensive Care, Faculty of Medicine, University of Debrecen, 4032 Debrecen, Hungary; siro.peter@med.unideb.hu; 2 Department of Pulmonology, Faculty of Medicine, University of Debrecen, 4032 Debrecen, Hungary; szabo-szucs.regina@med.unideb.hu (R.S.-S.); dudas.viktoria@med.unideb.hu (V.D.); horvath.ildiko@med.unideb.hu (I.H.); vasko.attila@med.unideb.hu (A.V.)

**Keywords:** COPD, cerebral vasoreactivity, acetazolamide, transcranial Doppler sonography

## Abstract

**Background:** COPD patients may be prone to cerebral small vessel disease resulting in perivascular white matter lesions and consequent cognitive decline. The pathophysiological background of these observations is not completely understood. It is hypothesized that COPD may involve systemic vascular dysfunction extending to the brain. The present study aimed to assess whether acetazolamide-induced cerebral vasoreactivity and cerebrovascular reserve capacity are impaired in patients with COPD. **Methods:** A total of 17 patients with COPD and 20 healthy control subjects underwent transcranial Doppler monitoring before and after IV administration of 15 mg/kgBW acetazolamide for 20 min. Cerebrovascular reactivity (CVR) was defined as a percent increase in blood flow velocity in the middle cerebral artery (MBFV) after acetazolamide. Cerebrovascular reserve capacity (CVRC) was defined as the maximal percent change in MBFV during the entire registration. **Results:** Administration of acetazolamide resulted in a slight decrease in pH and a mild increase in PaCO_2_ (both *p* < 0.001) in COPD patients. Absolute MBFV values were consequently higher, and pulsatility indices were lower in control subjects compared to those measured in patients with COPD. The CVR at different time points after acetazolamide and CVRC did not show any difference between COPD patients and control subjects. **Conclusions:** In the present study, in normocapnic mild and normocapnic moderate COPD patients, cerebrovascular reactivity is not impaired, indicating that in mild stages, cerebral arteriolar function is preserved. Further studies, using patient selection based on different severity stages of the disease, may show whether alteration of the cerebral arteriolar function is responsible for the white matter lesions and cognitive decline observed in severe COPD patients.

## 1. Introduction

Chronic obstructive pulmonary disease (COPD) represents one of the leading causes of death worldwide [1]. It is believed that COPD is the expression of a generalized inflammatory syndrome caused by significant exposure to external factors [2]. COPD and stroke share common risk factors, such as advancing age, smoking, socioeconomic status and air pollution. As a consequence, the incidence of stroke is increased in COPD patients by approximately 20% [3,4]. The affection of the brain in COPD is further supported by observations reporting on an association between impaired lung function, cerebral atrophy and white matter lesions of the brain parenchyma [5]. More recent studies using functional MRI reported on reduced white matter integrity and a widespread disturbance in functional activation of the gray matter, along with reduced cognitive performance in patients suffering from COPD [6,7]. The pathophysiological background of these observations is not completely understood; however, chronic low-grade systemic inflammation, hypoxia, hypercapnia and oxidative stress are the proposed factors for explaining it. It is also known that white matter lesions are believed to be caused by small vessel disease of the brain [8]. These small resistance arterioles are responsible for meeting the metabolic demands of the brain parenchyma. It was shown that the function of the cerebral microvessels is impaired in patients with white matter lesions. Their functions have been extensively studied using cerebral vasoreactivity and autoregulation tests in hypertension, diabetes mellitus and dementia [9,10,11]. There are several previous reports indicating that the function of these resistance arterioles may be affected in patients suffering from COPD, linking COPD to cognitive impairment and cerebrovascular disease. While the pulmonary consequences of COPD are well characterized, its cerebral vascular effects remain poorly understood.

Data on cerebral vasoreactivity tests as a surrogate marker of cerebral arteriolar function in patients suffering from chronic obstructive pulmonary disease are scarce and inconclusive. In line with this, the present study aimed to assess whether acetazolamide-induced cerebral vasoreactivity and cerebrovascular reserve capacity are impaired in patients with normocapnic, mild–moderate COPD.

## 2. Methods

This is a prospective case series of patients with previously verified chronic obstructive pulmonary disease treated at the Department of Pulmonology, University of Debrecen, as inpatients. The protocol of the study was approved by the local (Medical Ethics Committee of the University of Debrecen, DE RKEB 6268-2022, approval date: 22 December 2002) and national (Medical Research Council Ethics Committee for Clinical Pharmacology, registration number: BM/6408-1/2023, approval date:18 April 2023) ethical authorities. The study was registered prior to patient inclusion at the Clinical Trials registry (EUDRACT number: 2022-004076-41; registration date: 28 November 2022).

The criteria for COPD were defined using the Global Initiative for Chronic Obstructive Lung Disease (GOLD) criteria [12] in all patients.

Eligibility criteria: age between 50 and 75 years; ex-smokers (at least one year of interruption); and patients without exacerbation of the disease in the last two months. Exclusion criteria were as follows: use of home oxygen therapy, recent or ongoing theophylline therapy, cerebrovascular diseases or diseases affecting the cerebral vasculature (diabetes mellitus, severe hypertension, autoimmune vasculitides) in the previous history. All included patients were hospitalized COPD patients after stabilization of their recent exacerbations. Those patients having a temporal bone window not allow the insonation of the middle cerebral artery were also excluded.

Acetazolamide test results of non-COPD patients were used from a historical institutional database that aimed to determine the normal cerebrovascular reactivity values in healthy individuals in different age groups [13]. The test results of age and sex-matched controls were used for comparison.

Transcranial Color-Coded Duplex Sonography: The middle cerebral artery (MCA) was insonated using the 2 MHz sector probe of the Mindray M6 ultrasound device (Mindray, Shenzen, China) through the temporal bone. The vessel was localized using the color mode of the device, and blood flow velocities in the MCA were measured after angle correction at 45–50 mm depth. We have chosen acetazolamide as a vasodilatory stimulus because its administration and dosing are simple; furthermore, it is free of unpleasant side effects (such as fear of death) that may be observed during CO_2_ inhalation. It is also known that after administration of acetazolamide, it does not have any influence on systemic blood pressure; therefore, it allows testing the metabolic regulation of the cerebral arteriolar tone without affecting cerebral autoregulation. This vasodilatory stimulus has been widely used for testing cerebrovascular reactivity in different conditions that may affect the function of the cerebral small vessels [14]. It has been proven that a dose of 15 mg/kg BW corresponds to a supramaximal stimulus in cerebral vasoreactivity testing [14]. This explains why we used this dose. Systolic, diastolic and mean blood flow velocities and pulsatility indices were registered in the middle cerebral artery in the supine position of the patients. After registering the resting blood flow velocities, 15 mg/kg BW acetazolamide (XGEN Pharmaceuticals DJB, Inc., Horseheads, NY, USA) was injected slowly intravenously.

Cerebral blood flow velocity measurements were then repeated at 5, 10, 15 and 20 min after administration of acetazolamide (ACZ). In parallel with the transcranial Doppler measurements, arterial blood was sampled for blood gas analysis to determine the following parameters: pH, arterial pressure of oxygen (PaO_2_), carbon dioxide (PaCO_2_) and bicarbonate concentration (HCO_3_). These patients all had arterial lines allowing repeated sampling of arterial blood. In healthy control patients, serial arterial blood gas measurements were considered unethical, and, therefore, they were omitted.

### Calculation of Cerebrovascular Reactivity and Reserve Capacity

In line with previous vasoreactivity studies with acetazolamide, cerebrovascular reactivity (CVR) was defined as the percent increase in the cerebral blood flow in the middle cerebral artery at 5, 10, 15 and 20 min after administration of the drug [14]. CVR was calculated using the following equation:

CVR = (MBFV_acz_ − MBFV_rest_)/MBFV_rest_ × 100

where MBFV_acz_ is the mean blood flow velocity after ACZ at 5, 10, 15 and 20 min, and MBFV_rest_ is the mean blood flow velocity before administration of ACZ.

Cerebrovascular reserve capacity (CRC) was defined as the maximal percent change after administration of ACZ within the 20 min registration time and was calculated based on the following equation:

CRC = (MBFV_aczMAX_ − MBFV_rest_)/MBFV_rest_ × 100

where MBFV_aczMAX_ is the highest mean blood flow velocity after ACZ within the 20 min registration time, and MBFV_rest_ is the mean blood flow velocity before administration of ACZ.

Statistical Analysis: Before starting the study, we performed a power analysis to estimate the necessary number of included patients. We took the results of a previous acetazolamide test by Clivati [15] into account. Based on their data, cerebrovascular reserve capacity was 42 ± 9% in healthy subjects. We anticipated a decrease in cerebrovascular reserve capacity of 25% (which is half of the CRC decrease that was found in their study). Using an alpha of 0.05 and a power of 90%, 15 controls and 15 COPD patients had to be included in the present study. The parameters are reported as means ± standard deviations or medians and 25–75% interquartile ranges, depending on the results of the normality test. Parameters with normal distributions were compared between the two groups using the appropriate *t*-tests, while those with non-normal distributions were compared with Mann–Whitney tests. A *p* < 0.05 value was accepted as a statistically significant difference.

## 3. Results

A total of 17 patients with COPD and 20 healthy individuals were included. The most important clinical characteristics of the COPD patients are summarized in Table 1.

### 3.1. Results of Blood Gas Analysis Measurements

For better interpretation of the cerebral blood flow measurement results, we analyzed the changes in arterial blood gas parameters before and after administration of acetazolamide. The results are summarized in Table 2. Administration of acetazolamide resulted in a slight decrease in pH, a mild increase in PaCO_2_, and an improvement of PaO_2_, while bicarbonate levels were relatively stable throughout the 20 min registration period.

### 3.2. Results of Cerebral Blood Flow Velocity Measurements

Absolute cerebral blood flow velocity values were consequently higher, and pulsatility indices were lower in control subjects compared to those measured in patients with COPD. This difference could be observed both in the resting state and at all time points after administration of acetazolamide. The results are summarized in Table 3.

### 3.3. Cerebrovascular Reactivity (CVR) Measurements

**Figure 1:**
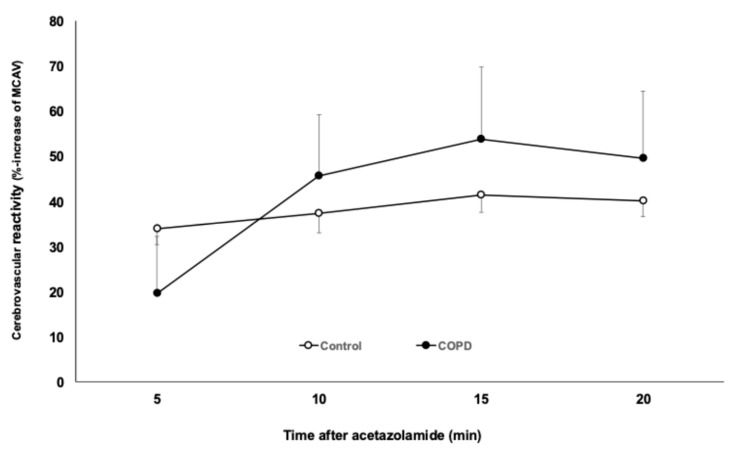
The percent increase in middle cerebral artery mean blood flow velocity (MCAV) in healthy control subjects and in COPD patients after administration of acetazolamide. Means ± standard errors are shown.

The percent increase in cerebral blood flow velocities at different time points after acetazolamide (cerebrovascular reactivity) did not show any difference between COPD patients and control subjects, suggesting a similar vasodilatory reaction of the cerebral arterioles. These values are detected in a middle-sized vessel of the circle of Willis, and the change in the cerebral blood flow velocity reflects the vasodilatory ability of the arterioles in the corresponding territory. It has to be noted that at 5 min, the median value of cerebrovascular reactivity was lower, indicating a slower reaction of the cerebral arterioles to the vasodilatory stimulus. In later phases of the acetazolamide test, the percent increase in the cerebral blood flow velocity was comparable between the control and COPD groups, suggesting that vasodilation of a similar magnitude occurred at the cerebral arteriolar level. The results are depicted in Figure 1.

We assessed the relationship between the actual blood gas analysis results after administration of acetazolamide and their relation to the measured cerebral vasoreactivity (the actual percent increase in cerebral blood flow velocity at different time points after the vasodilatory stimulus). The percent increase in cerebral blood flow velocity after acetazolamide was independent of the actual PaCO_2_ (Spearman: 0.07; *p* = 0.54) and the PaO_2_/PaCO_2_ ratio (Spearman: 0.22; *p* = 0.07). In contrast to this, a significant positive relationship could be found between PaO_2_ and the actual percent increase in cerebral blood flow velocity (Spearman: 0.32; *p* = 0.007).

### 3.4. Cerebrovascular Reserve Capacity

**Figure 2:**
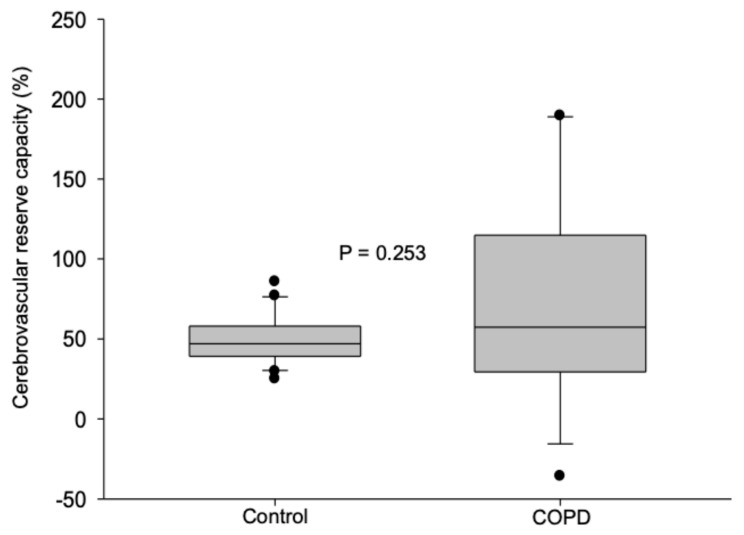
Cerebrovascular reserve capacity (= maximal percent change in the middle cerebral artery mean blood flow velocity) values in COPD patients and in healthy control subjects. Medians and 25–75% interquartile ranges are presented.

The maximal percent change in the middle cerebral artery mean blood flow velocity (= cerebrovascular reserve capacity) did not show any statistically significant differences between COPD patients and healthy subjects, suggesting that the maximal vasodilatory capacity of the cerebral arterioles is similar in the two groups. The results are shown in Figure 2. 

#### Relationship Between Cerebrovascular Reserve Capacity and Confounding Factors

The maximal response of the cerebral vasculature to the vasodilatory stimulus (cerebrovascular reserve capacity) did not show any relationship with the severity of COPD. Correlation analysis of FEV1 and cerebrovascular reserve capacity revealed a Spearman correlation coefficient of 0.09 and a *p*-value of 0.71. Similar to this, cerebrovascular reserve capacity showed no relationship with the Mini-Mental Score of COPD patients in this cohort (Spearman: −0.01, *p* = 0.95).

## 4. Discussion

In the present study, acetazolamide was used as a vasodilatory stimulus for assessing cerebral vasoreactivity and cerebrovascular reserve capacity in patients suffering from COPD. No difference was found between the time course of the cerebral vasodilatory effect (cerebral vasoreactivity) and the maximal vasodilatory effect of acetazolamide (cerebrovascular reserve capacity) in stable, normocapnic COPD patients. This may indicate that in this phase of the disease, the function of the cerebral resistance arterioles is not affected.

**Figure 3:**
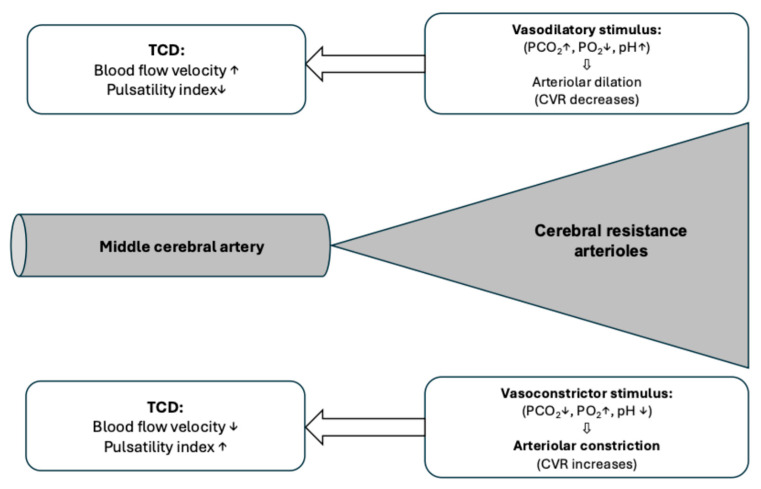
The concept of cerebrovascular reactivity testing using transcranial Doppler sonography. TCD = transcranial Doppler; PCO_2_ = partial pressure of CO_2_; PO_2_ = partial pressure of O_2_; CVR = cerebrovascular resistance. The site of the TCCD measurements is the middle cerebral artery, but the vasodilatory/vasconstrictor stimulus exerts its action on the arterioles of the corresponding vascular bed. The figure summarizes the action of different vasoactive stimuli and the consequent changes in the transcranial Doppler parameters.

The concept of cerebral vasoreactivity tests performed using transcranial Doppler sonography is demonstrated in Figure 3. The registration of Doppler parameters occurs in a mid-sized artery of the brain (usually the middle cerebral artery), but the vasodilatory or vasoconstrictor stimulus exerts its action by altering the diameter of the cerebral arterioles that are the resistance vessels of the brain. If a vasoconstrictor stimulus (acetazolamide, CO_2_-inhalation or breath holding) is applied during the vasoreactivity test, this decreases cerebrovascular resistance and, consequently, cerebral blood flow in the middle cerebral artery increases.

Acetazolamide has been widely used in the past decades for testing cerebral vasoreactivity in different conditions affecting the cerebral vasculature, such as hypertension, diabetes mellitus and sepsis [9,10,16]. It is the reversible inhibitor of carbonic anhydrase that catalyzes the following reaction: CO_2_+ H_2_O = HCO_3_^−^ + H^+^. This ubiquitous enzyme is expressed in different tissues, but the main vasodilatory effect is attributed to the carbonic anhydrase located at the surface of the erythrocytes [14,17]. As also demonstrated in the present study (Table 2), administration of acetazolamide slightly decreased the pH of the blood and increased the partial pressure of CO_2_. Repeated measures of ANOVA indicated a significant treatment mean effect of acetazolamide on the decrease in pH (*p* < 0.001), indicating that, in general, pH decreases after administration of acetazolamide. Note that after pairwise comparisons, pH decreased significantly only 10 min after acetazolamide compared to the resting value, and this difference continued to exist until the end of the measurements. Similar to this, acetazolamide administration exerted a significant time main effect on the partial pressure of CO_2_ during the measurements (*p* < 0.001). These effects using an intravenous administration are temporary and have a certain dynamics: during the first 5, 10 and 15 min after the administration, PaCO_2_ and, consequently, cerebral blood flow in the middle cerebral artery increased, reaching a plateau, and they started to become normalized 20 min after injecting the drug. These observations are in line with the results of previous acetazolamide tests performed in healthy volunteers [13]. This explains why cerebral vasoreactivity tests using transcranial Doppler sonography are performed for 20 min, and a combination of the maximal vasodilatory effect (cerebrovascular reserve capacity) and the dynamics of the vasodilatory response (cerebral vasoreactivity) is assessed. The cerebral vasodilatory effect of acetazolamide is dose-dependent. It has been shown that an intravenously administered dose of 1000 mg acetazolamide used in some previous studies may not have a maximal vasodilatory effect, and the suggested dose for cerebral vasoreactivity tests is 13–15 mg/kgBW [18]. This explains why we used a dose of 15 mg/kg body weight in the present study.

In contrast to our results, in a previous study by Clivati and co-workers [15], using a 10 mg/kg BW acetazolamide, a slightly decreased middle cerebral artery mean blood flow velocity change was observed in eight COPD patients compared to normal subjects. It has to be noted that resting cerebral blood flow velocities in that study were comparable in normal subjects and in COPD patients, whereas in our study, resting cerebral blood flow in COPD patients was lower. Another difference between this study and ours is the dose of the vasodilatory stimulus. Similar to our results, in the study of Hlavati and co-workers, COPD patients had lower baseline mean blood flow velocities in the middle cerebral artery than controls [19]. These authors also documented a decreased cerebral vasoreactivity in COPD patients using the breath-holding test. Note that abnormal reactivity was observed only in the groups of severe and very severe COPD patients, but not in mild and moderate cases. In a more recent study using CO_2_ inhalation, Corrêa and co-workers also documented reduced cerebrovascular reactivity in severe COPD patients [20]. This underscores the role of patient selection when assessing cerebrovascular responses in COPD patients and is supported by the observation of Yildiz and co-workers and Van den Ven and co-workers, who documented paradoxical responses of cerebral blood flow in stable COPD patients with chronic hypercapnia and hypoxia, and they found altered cerebrovascular responses in exacerbations of COPD [21,22]. As our patients represent the stable normocapnic COPD group, this may explain why we could not demonstrate differences in cerebral vasoreactivity compared to healthy subjects.

The limitation of the present study is the relatively small number of patients with mild or moderate COPD. Therefore, the lack of impaired vasoreactivity response cannot be generalized for more severe and exacerbation cases, and especially not for patients living with chronic hypercapnia. The methodological limitation is that transcranial Doppler sonography does not directly measure cerebral blood flow; only the cerebral blood flow velocities are proportional to changes in cerebral blood flow. We used acetazolamide as a vasodilatory stimulus, which is not a natural stimulation of the cerebral arterioles. However, other methods, such as CO_2_ inhalation or breath-holding test, may have side effects or may not be well standardized, as they require the cooperation of the patients.

In conclusion, in the present study, in mild and moderate normocapnic COPD patients, cerebrovascular reactivity and reserve capacity were not impaired compared to healthy control subjects, indicating that in the early stage of the disease, the function of the cerebral small vessels is not affected. It is conceivable that chronic inflammation, oxidative stress and cytokines associated with the pathomechanism of COPD in the early phase of the disease do not have a major effect on cerebral arteriolar function and hence cerebrovascular reactivity. Further studies, using patient selection based on different severities of the disease, will show whether alteration of the cerebral arteriolar function is responsible for the white matter lesions and the consequent cognitive disturbances observed in severe COPD patients.

## Figures and Tables

**Table 1:** Clinical characteristics and lung function test results of COPD patients. Means ± standard deviations are presented.

Parameter	Value
Female/Male	8/9
Age (years)	60.8 ± 4.9
BMI (kg/m^2^)	21.9 ± 5.6
Mini Mental Score	26.8 ± 3.1
FVC (L)	2.45 ± 0.97
FVC%	72.8 ± 19.1
FEV1 (L)	1.35 ± 0.74
FEV1%	48.2 ± 16.0
FEV1/FVC	52.6 ± 10.1
FEF25-75 (L/s)	0.85 ± 6.63
FEF 25-75%	27.2 ± 16.8

**Table 2:** Blood gas analysis results of COPD patients at rest and after IV administration of 15 mg/kg BW acetazolamide (ACZ). Means and standard deviations are presented.

	Rest	5 min After ACZ	10 min After ACZ	15 min After ACZ	20 min After ACZ
pH	7.43 ± 0.04	7.41 ± 0.04	7.41 ± 0.04	7.41 ± 0.04	7.41 ± 0.05
PaCO_2_ (mmHg)	42.5 ± 6.8	45.5 ± 7.6	45.2 ± 7.6	45.6 ± 7.6	45.0 ± 8.1
PaO_2_ (mmHg)	74.7 ± 16.9	81.5 ± 18.7	87.5 ± 22.4	87.6 ± 20.6	87.3 ± 20.0
HCO_3_ (mmol/L)	27.6 ± 2.6	27.6 ± 2.4	27.5 ± 2.4	27.6 ± 2.5	27.4 ± 2.4

**Table 3:** Cerebral blood flow velocities and pulsatility indices at rest and after administration of acetazolamide. Means and standard deviations and medians, and interquartile ranges are shown, as appropriate.

	Control (n = 20)	COPD (n = 17)	*p*-Value
Rest			
Systolic	85.9 ± 13.8	72.0 ± 24.8	*p* = 0.04
Diastolic	45.6 ± 8.8	28.5 ± 9.7	*p* ≤ 0.001
Mean	58.2 ± 12.0	44.2 ± 16.0	*p* < 0.01
PI	0.86 (0.64–1.0)	0.99 (0.9–1.0)	*p* < 0.05
5 min after acetazolamide			
Systolic	116.0 (98–126)	84.8 (64–109)	*p* < 0.01
Diastolic	61.9 ± 12.7	37.2 ± 15	*p* ≤ 0.001
Mean	77.8 ± 17.1	55.3 ± 22.8	*p* < 0.01
PI	0.8 ± 0.16	0.89 ± 0.13	*p* = 0.07
10 min after acetazolamide			
Systolic	119 (108–135)	89 (63–115)	*p* < 0.05
Diastolic	65 (52–77)	41 (29–52)	*p* < 0.01
Mean	79 (67–93)	57.3 (41.6–75.8)	*p* < 0.05
PI	0.71 ± 0.16	0.83 ± 0.17	*p* < 0.05
15 min after acetazolamide			
Systolic	126 (113–137)	91.1 (66.1–126.9)	*p* < 0.01
Diastolic	66 (57–70)	38.5 (28.1–53.5)	*p* < 0.01
Mean	82 (71–91)	56.0 (42.5–81.8)	*p* = 0.01
PI	0.76 ± 0.15	0.86 ± 0.12	*p* = 0.03
20 min afer acetazolamide			
Systolic	124 (116–132)	86.6 (68.1–118.2)	*p* < 0.01
Diastolic	64.7 ± 14.6	64.7 ± 14.6	*p* < 0.01
Mean	82 (69–93)	55 (40.4–75.8)	*p* = 0.01
PI	0.742 ± 0.141	0.85 ± 0.14	*p* < 0.05

Resting cerebral blood flow velocities in COPD patients did not show any statistically significant relationship with PaCO_2_ (Spearman coefficient: 0.26; *p* = 0.3) PaO_2_ (Spearman coefficient: 0.35; *p* = 0.16) and with PaO_2_/CO_2_ ratio (Spearman: 0.17; *p* = 0.51), indicating that actual blood gas parameters do not have a determining influence of cerebral blood flow velocities.

## Data Availability

The data are available from the corresponding author upon reasonable request.
